# Analysis of influencing factors for prognosis of patients with ventricular septal perforation: A single-center retrospective study

**DOI:** 10.3389/fcvm.2022.995275

**Published:** 2022-11-03

**Authors:** Ming-Xuan Duan, Xi Zhao, Shao-Lin Li, Jun-Zhong Tao, Bo-Yan Li, Xin-Guo Meng, Dong-Pu Dai, Yan-Yu Lu, Zhen-Zhen Yue, Yang Du, Zi-Ao Rui, Shuo Pang, Yuan-Hang Zhou, Guang-Rui Miao, Lin-Peng Bai, Qing-Yang Zhang, Xiao-Yan Zhao

**Affiliations:** ^1^Henan Key Laboratory of Hereditary Cardiovascular Diseases, Department of Cardiology, Cardiovascular Center, The First Affiliated Hospital of Zhengzhou University, Zhengzhou, China; ^2^Department of Cardiology, Chest Hospital of Henan Province, Zhengzhou, China

**Keywords:** acute myocardial infarction, ventricular septal rupture, percutaneous transcatheter closure, surgery, EuroSCORE II

## Abstract

**Background:**

Ventricular septal rupture (VSR) is a type of cardiac rupture, usually complicated by acute myocardial infarction (AMI), with a high mortality rate and often poor prognosis. The aim of our study was to investigate the factors influencing the long-term prognosis of patients with VSR from different aspects, comparing the evaluation performance of the Gensini score, Sequential Organ Failure Assessment (SOFA) score and European Heart Surgery Risk Assessment System II (EuroSCORE II) score systems.

**Methods:**

This study retrospectively enrolled 188 patients with VSR between Dec 9, 2011 and Nov 21, 2021at the First Affiliated Hospital of Zhengzhou University. All patients were followed up until Jan 27, 2022 for clinical data, angiographic characteristics, echocardiogram outcomes, intraoperative, postoperative characteristics and major adverse cardiac events (MACEs) (30-day mortality, cardiac readmission). Cox proportional hazard regression analysis was used to explore the predictors of long-term mortality.

**Results:**

The median age of 188 VSR patients was 66.2 ± 9.1 years and 97 (51.6%) were males, and there were 103 (54.8%) patients in the medication group, 34 (18.1%) patients in the percutaneous transcatheter closure (TCC) group, and 51 (27.1%) patients in the surgical repair group. The average follow-up time was 857.4 days. The long-term mortality of the medically managed group, the percutaneous TCC group, and the surgical repair group was 94.2, 32.4, and 35.3%, respectively. Whether combined with cardiogenic shock (OR 0.023, 95% CI 0.001–0.054, *P* = 0.019), NT-pro BNP level (OR 0.027, 95% CI 0.002–0.34, *P* = 0.005), EuroSCORE II (OR 0.530, 95% CI 0.305–0.918, *P* = 0.024) and therapy group (OR 3.518, 95% CI 1.079–11.463, *P* = 0.037) were independently associated with long-term mortality in patients with VSR, and this seems to be independent of the therapy group. The mortality rate of surgical repair after 2 weeks of VSR was much lower than within 2 weeks (*P* = 0.025). The cut-off point of EuroSCORE II was determined to be 14, and there were statistically significant differences between the EuroSCORE II < 14 group and EuroSCORE II≥14 group (HR = 0.2596, 95%CI: 0.1800–0.3744, Logrank *P* < 0.001).

**Conclusion:**

Patients with AMI combined with VSR have a poor prognosis if not treated surgically, surgical repair after 2 weeks of VSR is a better time. In addition, EuroSCORE II can be used as a scoring system to assess the prognosis of patients with VSR.

## Introduction

Ventricular septal rupture (VSR) is a type of heart rupture. Together with wall rupture and papillary muscle rupture, VSR is a serious and fatal mechanical complication of acute myocardial infarction (AMI), which often occurs within 1–2 weeks after AMI (1–3). This is usually due to the influx of a large number of neutrophils into the necrotic area, which releases enzymes after apoptosis, accelerating the destruction of the infarcted myocardium and resulting in ventricular septal perforation (4). When AMI patients are combined with VSR, the mortality rate is upwards of 90%. (5). It is currently believed that female, old age, combined hypertension, fresh infarction, anterior MI are the risk factors for AMI complicated by VSR (6–9). For VSR, conservative medical treatment, percutaneous transcatheter closure (TCC) or surgical repair are usually adopted clinically. The mortality rates of patients with medical treatment alone were extremely high (10). Hence, the European Society of Cardiology (ESC) and the American College of Cardiology Foundation/American Heart Association (ACCF/AHA) guidelines for ST-segment elevation myocardial infarction (STEMI) propose surgical closure as the standard of care (11, 12). But at present, there is no clear standard for the selection of operation timing. Studies have shown that the difficulty of the procedure, as well as the postoperative mortality and morbidity, all decline significantly in elective surgery compared to immediate surgery (13). Therefore, allowing time for recovery and healing prior to surgery is often encouraged (14). On the other hand, since elective surgery is often performed in patients with relatively stable VSR, including patients who were eventually hemodynamically stabilized by mechanical circulatory support (MCS), the difference in outcome between immediate and elective surgery may also be an indication of survival bias (15). In addition, the interventricular communication may expand in a considerable number of patients while waiting for intervention, resulting in increased right ventricular load and shunt fraction (16). Based on this, the 2013 AHA guidelines for STEMI recommended emergency surgical repair for all patients with VSR, even in hemodynamically stable patients (11).

In recent years, transcatheter technology have become increasingly sophisticated and can be used as an alternative to surgical closure of VSR for subacute and chronic VSR (17, 18). In addition, there are many improved new surgical methods, but the effect is not very clear, and the repeatability is low (19, 20). Three commonly used scoring systems were chosen for this study to investigate their relationship with survival in VSR patients. The Gensini score is one of the most widely used scoring systems to indicate the outcome of coronary angiography (21). The Sequential Organ Failure Assessment (SOFA) score summarizes and measures the severity of organ system dysfunction and failure, and is primarily used to assess the acute morbidity of critical conditions (22, 23). European Heart Surgery Risk Assessment System II (EuroSCORE II) is a widely used cardiac surgery scoring system. It can accurately assess preoperative risk, predict in-hospital mortality in postoperative patients, which is essential for providing treatment options and improving quality of care and patient prognosis (24, 25).

In our single-center retrospective study, we wanted to evaluate the prognostic factors of VSR patients from multiple perspectives, to compare the effects of conservative medical treatment, percutaneous TCC and surgical repair on the long-term prognosis of patients, and to clarify the more appropriate timing of surgical closure. We will calculate the Gensini, SOFA and EuroSCORE II score of all patients and analyze the relationship between the scores and long-term prognosis of VSR patients, aiming to provide useful information for the management and treatment of clinical VSR patients.

## Methods

### Study design and population

This was an analysis of retrospectively collected data including all adult (> 18 years of age) patients with a postinfarction VSR admitted to First Affiliated Hospital of Zhengzhou University between Dec 9, 2011 and Nov 21, 2021. Eligible patients were mainly those with evidence of VSR after MI:(a) a systolic murmur was heard between the third and fourth ribs on the left side on auscultation (b) transthoracic echocardiogram (TTE) confirms a left-to-right shunt and (c) left ventriculography showed contrast shunting from left to right (26). Exclusion criteria: VSR secondary to the presence of congenital heart disease; rheumatic heart disease; combined with cardiomyopathy, such as hypertrophic cardiomyopathy, etc.; known tumor or systemic disease (such as lupus erythematosus, nephrotic syndrome, etc.); serious infection; acute trauma; the patient's prognosis is not known by reviewing the medical records or making follow-up phone calls, that is, the patient is missed. Our study was approved by the ethical committee of the First Affiliated Hospital of Zhengzhou University (Approved No. of ethic committee 2022-KY-0041) and medical research involving human subjects in this study is in accordance with the ethical principles set forth in the Declaration of Helsinki (2013). In addition, written informed consent was obtained from each participant before we conducted the study.

### Data collection

From Dec 9, 2011 to Nov 21, 2021, a total of 193 patients with VSR were admitted to the First Affiliated Hospital of Zhengzhou University. All these patients were screened according to our inclusion criteria and exclusion criteria. Finally, we excluded 5 patients, one younger than 18 years old, one with congenital heart disease, one with myocardial infarction combined with stress myocarditis septal perforation, and two patients or their families could not be contacted through follow-up phone calls. Ultimately, 188 patients with VSR were enrolled in our study.

We obtained the clinical data of patients during their stay by querying the medical record system of our hospital, and received the long-term outcomes through telephone follow-up with the 188 VSR patients individually by trained professionals.

We retrieved demographic and clinical data on 188 patients with VSR, including age, sex, comorbidities, whether to transfer from other hospitals, MI staging, MI to VSR time, Killip classification, laboratory values upon ED admission (within 24 h), therapeutic interventions, Gensini score, SOFA score and EuroSCORE II score.

We divided 188 VSR patients into medication group, percutaneous TCC group and surgical repair group according to treatment modalities. The three groups were then further divided into survival group and death group according to survival at the end of follow-up.

Angiographic characteristics of VSR patients was also recorded in our statistics, which included the following information: the count and the type of lesion blood vessels, left main occlusion or not, with or without 100% vascular occlusion [100% occlusion refers to complete occlusion of one or more vessels in the left anterior descending (LAD), left circumflex (LCX), and right coronary arteries (RCA)]. We eventually collected angiography characteristics from 91 VSR patients because some patients were too critical for a coronary angiography, or patients underwent angiography outside our hospital but we could not obtain the results.

We also collected information about echocardiogram outcomes of VSR patients. All 188 patients underwent at least one TTE after admission, and we recorded the results within 24 h after perforation in each patient. These include MI territory (only patients with a single-site MI were counted), multiple MI or not, extensive anterior MI or not, left ventricle ejection fraction (LVEF), left ventricular end-diastolic dimension (LVEDD), maximum rupture size (defined as the maximum value of the defect diameter in mm, measured by transthoracic ultrasound), multiple VSR or not, whether combined with ventricular aneurysm, the number of patients with pulmonary artery pressure (PAP) over 60, VSR location (divided as apical, anterior, posterior or anterior + posterior) and whether the site of VSR is the posterior segment.

To further explore the relationship between preoperative and intraoperative characteristics and the long-term mortality of patients in surgical repair group, we recorded operation-related information from the medical record system, which mainly includes preoperative NT-pro BNP levels, EuroSCORE II, the occurrence of cardiogenic shock, surgical status (surgery within 2 weeks of septal perforation is defined as emergency surgery, otherwise it is elective surgery), and operation consuming time, surgical incision (including ventricular aneurysm incision, left ventricular incision, right atrium tricuspid valve incision and aortic root incision), surgical repair method [defined as direct suture, patch and surgical repair combining an occluder and a patch (SurCOP)], whether a coronary artery bypass graft (CABG) was also performed and the number and type of graft vessels, and whether the patient had a ventricular aneurysm resection and valvuloplasty (27).

After the percutaneous TCC or surgical repair, ventilator usage time, operation outcomes, length of stay in the hospital and the intensive care unit (ICU), post-operative residual shunt (The TTE results within 24 h after operation were taken as the standard), additional complications [e.g., renal failure requiring continuous renal replacement therapy (CRRT), postoperative blood transfusion and low cardiac output syndrome (LCOS)], readmission (defined as readmission due to cardiogenic disease), 30-day and long-term mortality were recorded.

### Study outcomes and definitions

The primary outcome was long-term mortality, which was defined as overall mortality during the follow-up period. Secondary outcomes are major adverse cardiac events (MACEs). We defined MACEs as 30-day mortality and readmission due to cardiogenic disease.

The definitions of the variables in this study are consistent with the cardiovascular data criteria (28). Cardiogenic shock is defined as sustained hypotension (systolic blood pressure < 90 mm Hg) with decreased cardiac index (< 1.8 l/min/m^2^) caused by cardiac blood displacement failure (29). There are three main methods for VSR surgical repair in our hospital: Daggett's procedure, David's procedure and SurCOP. Daggett's procedure removes the infarcted myocardium and closes the perforation directly using a patch (infarct excision), while David's procedure uses a pericardial slice to isolate the infarcted myocardium (infarct exclusion); David's procedure has better results than Daggett's procedure, and David's infarct zone isolation method is now mainly used to treat VSR (30, 31). While the third method called SurCOP is a new surgical technique, rupture was closed with a patent ductus arteriosus (PDA) occluder along with a bovine pericardial patch (32). In addition, there are four main types of surgical incisions to expose the perforation, which are: ventricular aneurysm incision, left ventricular incision, right atrium incision and aortic root incision. The incision of the left ventricle is parallel to the anterior descending or posterior descending artery. In some cases, the right atrium was cut to expose the tricuspid valve, and then the perforation site was exposed. When combined with ventricular aneurysm, incision of ventricular aneurysm is usually selected to expose perforation. While aortic root incisions are less common.

### Statistical analyses

Data were analyzed using IBM SPSS Statistics 25.0 (Armonk, NY: IBM Corp.) and GraphPad Prism v9.0 (GraphPad Software, La Jolla, CA, USA), using mean ± standard deviation (SD) to express if normal distribution continuous variables, median (25th−75th percentiles) if skew distribution continuous variables, and count (percentage) if categorical variables. The Shapiro-wilk test was used to verify that the continuous variables were normally distributed. Differences between groups were assessed using the Chi-square test or Fisher's exact test (when at least an expected value in a cell is < 5) for categorical variables and two independent sample *t*-test, or one-way analysis of variance (ANOV A) for continuous variables, as appropriate. We analyzed the influencing factors or predictors of prognosis of VSR patients by binary Logistic regression, the indicators entered into the regression analysis were as follows: sex, MI to VSR time, history of cerebral infarction, malignant arrhythmia, cardiogenic shock, lactic acid, white blood cells (WBC), hemoglobin, alanine transaminase (ALT), creatinine, glucose, n-terminal pro b-type natriuretic peptide (NT-pro BNP), cardiac troponin I (cTnI), LVEF, EuroSCORE II, SOFA score and therapy group. Levels of NT-pro BNP was normalized by log10 transformation. Estimation chart reflecting the difference in timing of surgery between the survivor and death groups in patients with surgically repaired VSR. Using the receiver operating characteristic (ROC) curve and the area under the curve (AUC) to evaluate the discriminative ability of EuroSCORE II, SOFA score, etc. to predict the prognosis of VSR patients, and calculate the sensitivity, specificity and 95% confidence interval (CI) of AUC. The best prediction threshold for each variable was calculated using the Youden's index. Kaplan–Meier survival curves and the Log-rank test were used to identify significant relationships between whether the EuroSCORE II was greater than the optimal prediction threshold and long-term mortality. The results are expressed as hazard ratio (HR) and 95% CI. Two-tailed *P* < 0.05 was considered statistically significant.

## Results

### Results of recruitment

**Figure 1:**
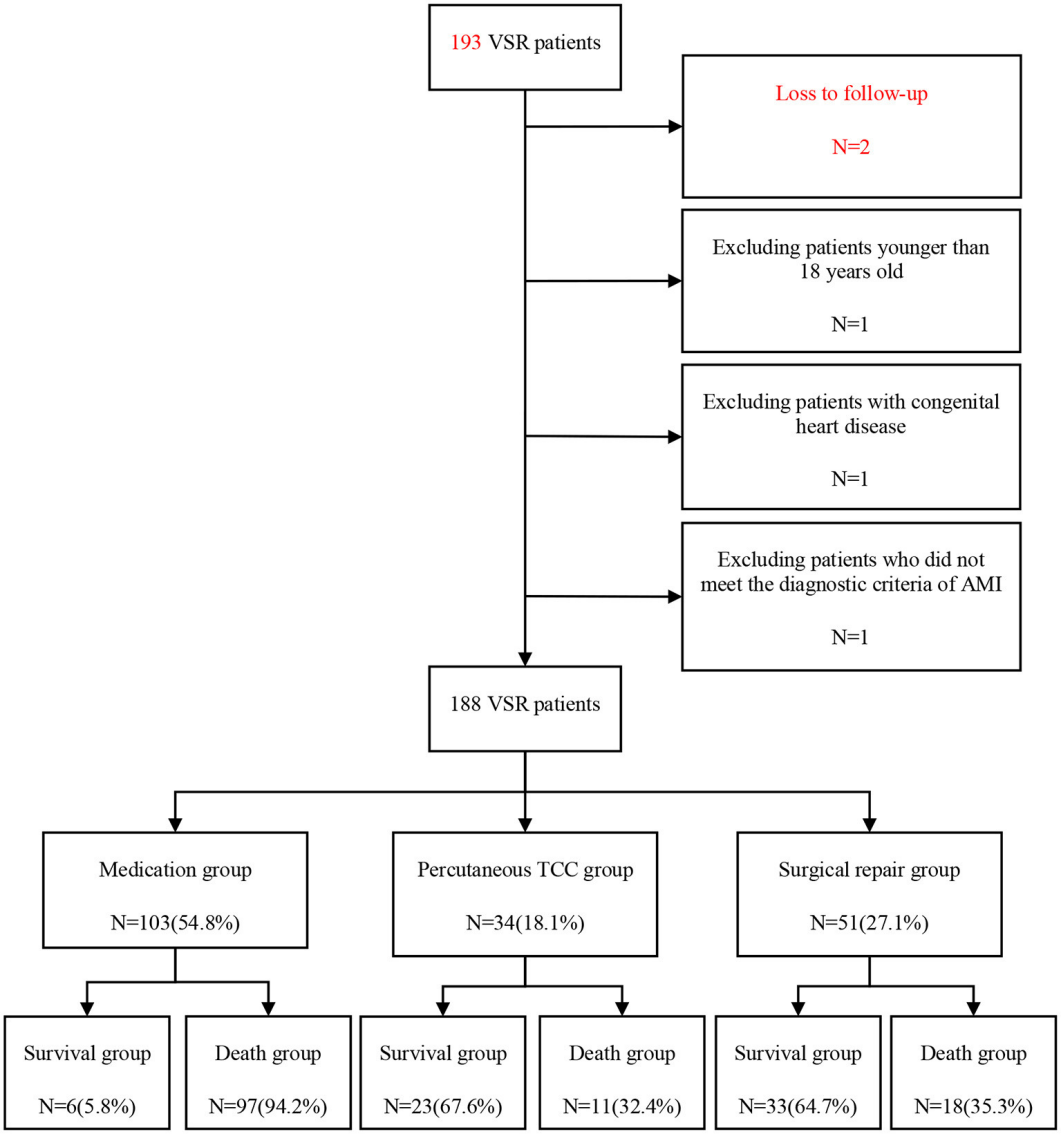
Flow diagram of patient inclusion and exclusion. VSR, ventricular septal rupture; AMI, acute myocardial infarction; TCC, transcatheter closure.

From 9 December 2011 to 21 November 2021, 193 VSR patients admitted to our hospital were screened, five patients were excluded from the analysis according to the predetermined criteria. After exclusion, a total of 188 VSR patients were enrolled in this study. Our follow-up date ended on 27 January 2022, the longest follow-up time was 3,702.1 days, and the shortest was 0.1 days, the median follow-up time was 17.0 days. Considering the bias caused by the short survival time of the patients in the medication group, we analyzed the follow-up time of the percutaneous TCC group and the surgical repair group, and the results showed that the median follow-up time was 575.0 days (25th−75th percentile:25.0–1374.5 days) [The average follow-up time was 857.4 days]. All patients were divided into three groups according to the treatment modalities, among which, there were 103 (54.8%) patients in the medication group, 34 (18.1%) patients in the percutaneous TCC group, and 51 (27.1%) patients in the surgical repair group. The enrollment and clinical grouping for VSR patients are shown in the flow diagram in Figure 1.

### Clinical characteristics

Characteristics of the study population are shown in Table 1; median age was 66.2±9.1 years and 97 (51.6%) were males. Through analysis, we found that compared with the survival group, the proportion of females in the death group was larger (69, 54.8%), and there were more patients with previous cerebral infarction (27, 21.4%), malignant arrhythmia (52, 41.3%), cardiogenic shock (46, 36.5%), preoperative cardiac arrest (12, 9.5%), renal failure (76, 60.3%) and multiple organ dysfunction syndrome (MODS) (23, 18.3%), shorter MI to VSR time (5.3±6.1), and higher Killip classification (4: 102, 81%) in the death group. In terms of laboratory values upon ED admission, hemoglobin [122.1 (109.0, 137.0)] and albumin [35.4 (33.0, 38.4)] in the death group were lower, while lactic acid levels [2.8 (1.7, 6.0)], WBC [13.7 (10.8, 16.7)], total bilirubin [20.7 (13.1, 30.0)], uric acid (499.0±280.4), creatinine (180.6±161.1), ALT (687.6±1,240.5), aspartate transaminase (AST) [202.0 (54.0, 5,054.0)], glucose (10.9±10.2), NT-pro BNP (15,057.1±11,620.3), and cTnI (8.4±17.2) were higher. According to the therapy groups, the proportion of patients treated with medication was highest in the death group [medication group (97, 77.0%) vs. percutaneous TCC group (11, 8.7%) vs. surgical repair group (18, 14.3%), *P* < 0.001]. And there was no significant survival difference between the percutaneous TCC and surgical repair group (*P* = 0.779). SOFA score [death group (14.3±5.3) vs. survival group (10.0±3.0), *P* < 0.001] and EuroSCORE II [death group (16.0±2.3) vs. survival group (12.5±2.0), *P* < 0.001] in the death group were significantly higher than those in the survival group, and more patients in the death group required intra-aortic balloon pump (IABP) (43, 34.1%), CRRT (21, 16.7%) and central venous catheter (CVC) (70, 55.6%) treatment. In addition, the proportion of IABP difficult to offline in the death group was higher (39, 90.7%).

**Table 1:** Clinical data of VSR patients stratified by different prognosis.

**Clinical data**	**Grouping**	**Total number of cases** **(*n* = 188)**	**Survival group** **(*n* = 62)**	**Death group** **(*n* = 126)**	**χ^2^/t value/Z** **value**	***P* value**
**Demographic characteristics**						
Age, (years,$\bar{\chi }$ ±s)		66.2 ± 9.1	64.7 ± 8.6	66.9 ± 9.3	1.529	0.128
Sex, [n (%)]	Male	97 (51.6%)	40 (64.5%)	57 (45.2%)	6.183	**0.013**
	Female	91 (48.4%)	22 (35.5%)	69 (54.8%)		
Age over 60, [n (%)]		148 (78.7%)	45 (72.6%)	103 (81.7%)	2.084	0.149
**Comorbidities**						
Smoking, [n (%)]		47 (25.0%)	20 (32.3%)	27 (21.4%)	2.599	0.107
Drinking, [n (%)]		30 (16.0%)	12 (19.4%)	18 (14.3%)	0.796	0.372
Hypertension, [n (%)]		101 (53.7%)	32 (51.6%)	69 (54.8%)	0.166	0.684
History of hypertension, (years, $\bar{\chi }$ ± s)		5.6 ± 8.1	5.1 ± 8.7	5.8 ± 7.8	0.573	0.568
Diabetes, [n (%)]		62 (33.0%)	20 (32.3%)	42 (33.3%)	0.022	0.883
History of diabetes, (years, $\bar{\chi }$ ± s)		2.6 ± 5.0	2.3 ± 4.5	2.7 ± 5.2	0.434	0.664
Hyperlipidemia, [n (%)]	With	20 (12.8%)	7 (13.0%)	13 (12.7%)	0.001	0.969
	Without	136 (87.2%)	47 (87.0%)	89 (87.3%)		
History of cerebral infarction, [n (%)]		32 (17.0%)	5 (8.1%)	27 (21.4%)	5.254	**0.022**
History of myocardial infarction, [n (%)]		17 (9.0%)	6 (9.7%)	11 (8.7%)	0.045	0.831
History of angioplasty, [n (%)]		8 (4.3%)	5 (8.1%)	3 (2.4%)	2.047	0.152
Prior fibrinolysis therapy, [n (%)]		14 (7.4%)	4 (6.5%)	10 (7.9%)	0.005	0.945
Pericardial effusion, [n (%)]		69 (36.7%)	24 (38.7%)	45 (35.7%)	0.160	0.689
Pleural effusion, [n (%)]		47 (25.0%)	17 (27.4%)	30 (23.8%)	0.289	0.591
III AVB, [n (%)]		4 (2.1%)	0 (0.0%)	4 (3.2%)	0.775	0.379
Malignant arrhythmia, [n (%)]		57 (30.3%)	5 (8.1%)	52 (41.3%)	21.686	**< 0.001**
Cardiogenic shock, [n (%)]		47 (25.0%)	1 (1.6%)	46 (36.5%)	26.985	**< 0.001**
Preoperative cardiac arrest, [n (%)]		12 (6.4%)	0 (0.0%)	12 (9.5%)	4.814	**0.028**
Right heart failure, [n (%)]		45 (23.9%)	10 (16.1%)	35 (27.8%)	3.097	0.078
Renal failure, [n (%)]		91 (48.4%)	15 (24.2%)	76 (60.3%)	21.712	**< 0.001**
MODS, [n (%)]		23 (12.2%)	0 (0.0%)	23 (18.3%)	12.895	**< 0.001**
**The clinical situation**						
Transferred from elsewhere, [n (%)]		96 (51.1%)	32 (51.6%)	64 (50.8%)	0.011	0.916
	Acute phase	181 (96.3%)	57 (91.9%)	124 (98.4%)		
MI staging	Subacute phase	2 (1.1%)	2 (3.2%)	0 (0.0%)	5.897	0.052
	Remote infarct	5 (2.7%)	3 (4.8%)	2 (1.6%)		
MI to VSR time, (d,$\bar{\chi }$ ± s)		6.2 ± 6.9	8.0 ± 7.9	5.3 ± 6.1	−2.608	**0.010**
Killip classification, [n (%)]	1	1 (0.5%)	0 (0.0%)	1 (0.8%)	36.973	**< 0.001**
	2	35 (18.6%)	23 (37.1%)	12 (9.5%)		
	3	27 (14.4%)	16 (25.8%)	11 (8.7%)		
	4	125 (66.5%)	23 (37.1%)	102 (81.0%)		
**Laboratory data**						
Lactic acid, [mmol/L, M (P25, P75)]		2.0 (1.4, 4.6)	1.4 (0.9, 1.9)	2.8 (1.7, 6.0)	−5.448	**< 0.001**
WBC, [*10^9^/L, M (P25, P75)]		11.4 (7.9, 15.1)	8.4 (6.4, 11.9)	13.7 (10.8, 16.7)	−5.907	**< 0.001**
Hemoglobin, [g/L, M (P25, P75)]		126.0 (114.0, 137.0)	131.0 (122.2, 137.2)	122.1 (109.0, 137.0)	−2.293	**0.022**
Albumin, [g/L, M (P25, P75)]		36.0 (33.5, 38.9)	36.8 (33.7, 40.2)	35.4 (33.0, 38.4)	−2.050	**0.040**
Total bilirubin, [μmmol/L, M (P25, P75)]		15.0 (10.9, 27.5)	12.8 (9.1, 16.0)	20.7 (13.1, 30.0)	−3.712	**< 0.001**
HDL, (mmol/L,$\bar{\chi }$ ± s)		0.9 ± 0.3	0.9 ± 0.3	0.9 ± 0.4	0.804	0.423
LDL, (mmol/L,$\bar{\chi }$ ± s)		2.3 ± 0.9	2.2 ± 0.8	2.3 ± 1.0	0.661	0.510
Uric acid, (μmol/L,$\bar{\chi }$ ± s)		448.8 ± 260.9	347.6 ± 179.4	499.0 ± 280.4	3.780	**< 0.001**
Creatinine, (μmol/L,$\bar{\chi }$ ± s)		151.8 ± 139.6	93.2 ± 37.5	180.6 ± 161.1	4.140	**< 0.001**
eGFR, (mL/min/1.73m^2^,$\bar{\chi }$ ± s)		56.3 ± 33.9	72.6 ± 22.5	47.2 ± 35.7	−4.820	**< 0.001**
ALT, (U/L,$\bar{\chi }$ ± s)		514.6 ± 1,083.8	171.5 ± 535.1	687.6 ± 1,240.5	3.078	**0.002**
AST, [U/L, M (P25, P75)]		91.0 (27.8, 557.8)	26.5 (29.0, 45.0)	202.0 (54.0, 5,054.0)	−6.724	**< 0.001**
Glucose, (mmol/L,$\bar{\chi }$ ± s)		9.9 ± 8.7	7.9 ± 3.8	10.9 ± 10.2	2.220	**0.028**
HbA1C, (%,$\bar{\chi }$ ± s)		7.3 ± 1.8	7.7 ± 2.0	7.1 ± 1.7	−1.552	0.123
NT-pro BNP, (pg/ml,$\bar{\chi }$ ± s)		12,023.4 ± 11,136.1	5,750.3 ± 6,615.8	15,057.1 ± 11,620.3	5.715	**< 0.001**
CTnI, (mmol/L,$\bar{\chi }$ ± s)		6.4 ± 14.6	2.3 ± 4.5	8.4 ± 17.2	2.678	**0.008**
	Medication group	103 (54.8%)	6 (9.7%)	97 (77.0%)		
**Therapy group**	Percutaneous TCC group	34 (18.1%)	23 (37.1%)	11 (8.7%)	76.074	**< 0.001**
	Surgical repair group	51 (27.1%)	33 (53.2%)	18 (14.3%)		
**Therapeutic interventions**						
ECMO support, [n (%)]		11 (5.9%)	2 (3.2%)	9 (7.1%)	0.556	0.456
IABP support, [n (%)]		50 (26.6%)	7 (11.3%)	43 (34.1%)	11.100	**0.001**
IABP difficult to offline, [n (%)]		40 (80.0%)	1 (14.3%)	39 (90.7%)	17.452	**< 0.001**
CRRT, [n (%)]		22 (11.7%)	1 (1.6%)	21 (16.7%)	9.113	**0.003**
CVC, [n (%)]		84 (44.7%)	14 (22.6%)	70 (55.6%)	18.280	**< 0.001**
Vasoactive drug, [n (%)]		188 (100.0%)	62 (100.0%)	126 (100.0%)	-	-
**Severity score**						
Gensini score, ($\bar{\chi }$ ± s)		66.4 ± 39.2	63.6 ± 37.3	69.2 ± 41.3	0.671	0.504
SOFA score, ($\bar{\chi }$ ± s)		12.8 ± 5.1	10.0 ± 3.0	14.3 ± 5.3	5.950	**< 0.001**
EuroSCORE II, ($\bar{\chi }$ ± s)		14.8 ± 2.7	12.5 ± 2.0	16.0 ± 2.3	9.910	**< 0.001**

^*^VSR, ventricular septal rupture; AVB, atrioventricular block; MODS, multiple organ dysfunction syndrome; AMI, acute myocardial infarction; WBC, white blood cells; HDL, high-density lipoprotein; LDL, low-density lipoprotein; eGFR, estimated glomerular filtration rate; ALT, alanine transaminase; AST, aspartate transaminase; HbA1C, Hemoglobin A1C; NT-pro BNP, N-terminal pro b-type natriuretic peptide; CTnI, cardiac troponin I; TCC, transcatheter closure; ECMO, extracorporeal membrane oxygenation; IABP, intra-aortic balloon pump; CRRT, continuous renal replacement therapy; CVC, central venous catheter. SOFA, sequential organ failure assessment; EuroSCORE II, European heart surgery risk assessment system II. P values in bold meant significantly different (P < 0.05).

### Angiographic and echocardiograms outcomes

Angiographic characteristics and echocardiogram outcomes of 188 VSR patients are shown in Tables 2, 3. There was no significant difference in angiographic characteristics between survival and death groups. In the light of the echocardiogram outcomes, patients in the death group had lower LVEF (48.2 ± 10.0), and the proportion of posterior perforation was lower (15, 11.9%).

**Table 2:** Angiographic characteristics of VSR patients stratified by different prognosis.

**Angiographic characteristics [n (%)]**	**Total number of cases (*n* = 91)**	**Survival group (*n* = 45)**	**Death group** **(*n* = 46)**	**χ^2^ value**	***P* value**
One-branch lesions	12 (13.2%)	8 (17.8%)	4 (8.7%)	2.249	0.325
Two-branch lesions	27 (29.7%)	11 (24.4%)	16 (34.8%)		
Three-branch lesions	52 (57.1%)	26 (57.8%)	26 (56.5%)		
LM	8 (8.8%)	3 (37.5%)	26 (62.5%)	0.114	0.736
LM+ three-branch lesions	6 (6.6%)	3 (50.0%)	3 (50.0%)	0.000	1.000
LAD	9 (9.9%)	5 (11.1%)	4 (8.7%)	5.919	0.314
RCA	3 (3.3%)	3 (6.7%)	0 (0.0%)		
LCX	0 (0.0%)	0 (0.0%)	0 (0.0%)		
LAD+LCX	14 (15.4%)	7 (15.6%)	7 (15.2%)		
LAD+RCA	11 (12.1%)	4 (8.9%)	7 (15.2%)		
LCX+RCA	2 (2.2%)	0 (0.0%)	2 (4.3%)		
LAD+RCA+LCX	52 (57.1%)	26 (57.8%)	26 (56.5%)		
100% occlusion	40 (44.0%)	18 (45.0%)	22 (55.0%)	0.566	0.452

^*^VSR, ventricular septal rupture; LM, left main; LAD, left anterior descending branch; RCA, right coronary artery; LCX, left circumflex branch; 100% occlusion means complete occlusion in one or more of the three blood vessels.

**Table 3:** Echocardiogram outcomes of VSR patients stratified by different prognosis.

**Echocardiogram outcomes**	**Grouping**	**Total number of cases** **(*n* = 188)**	**Survival group** **(*n* = 62)**	**Death group** **(*n* = 126)**	**χ^2^/t value/Z value**	***P* value**
MI territory, [n (%)]	Anterior	40 (23.7%)	18 (33.3%)	22 (19.1%)	6.426	0.093
	Extensive anterior	81 (47.9%)	20 (37.0%)	61 (53.0%)		
	Inferior	34 (20.1%)	13 (24.1%)	21 (18.3%)		
	Anterior septal	14 (8.3%)	3 (5.6%)	11 (9.6%)		
Multiple MI, [n (%)]	With	19 (10.1%)	8 (12.9%)	11 (8.7%)	0.797	0.372
	Without	169 (89.9%)	54 (87.1%)	115 (91.3%)		
Extensive anterior MI, [n (%)]	With	100 (53.2%)	28 (45.2%)	72 (57.1%)	2.396	0.122
	Without	88 (46.8%)	34 (54.8%)	54 (42.9%)		
LVEF, (%, $\bar{\chi }$ ± s)		49.2 ± 10.0	51.2 ± 9.8	48.2 ± 10.0	−1.979	**0.049**
LVEDD, (mm, $\bar{\chi }$ ± s)		52.6 ± 6.7	53.3 ± 6.4	52.0 ± 6.9	−0.911	0.365
Maximum rupture size, [mm, M (P25, P75)]		8 (5, 11)	7.0 (5.4, 11.8)	8.0 (5.0, 11.3)	−0.036	0.972
Multiple VSR, [n (%)]		9 (4.8%)	4 (6.5%)	5 (4.0%)	0.149	0.699
Combined with ventricular aneurysm, [n (%)]		94 (50.0%)	36 (58.1%)	58 (46.0%)	2.407	0.121
PAP over 60, [n (%)]		15 (8.0%)	8 (12.9%)	7 (5.6%)	2.137	0.080
VSR location, [n (%)]	Apical	108 (57.4%)	32 (51.6%)	76 (60.3%)	5.595	0.109
	Anterior	50 (26.6%)	15 (24.2%)	35 (27.8%)		
	Posterior	29 (15.4%)	15 (24.2%)	14 (11.1%)		
	Anterior+ posterior	1 (0.5%)	0 (0.0%)	1 (0.8%)		
Posterior rupture, [n (%)]	With	30 (16.0%)	15 (24.2%)	15 (11.9%)	4.679	**0.031**
	Without	158 (84.0%)	47 (75.8%)	111 (88.1%)		

^*^VSR, ventricular septal rupture; MI, myocardial infarction; LVEF, left ventricle ejection fraction; LVEDD, left ventricular end-diastolic dimension; PAP, Pulmonary artery pressure; MI territory refers to the location classification of one-site MI. P values in bold meant significantly different (P < 0.05).

### Intraoperative characteristics of surgical repair group

Intraoperative characteristics of VSR patients in surgical repair group are shown in Table 4. There was a total of 51 patients in the surgical repair group, emergency operation was performed in 18 patients, and elective operation was performed in the remaining 33 patients. Mortality rates were higher for emergency surgery than for elective surgery (*P* = 0.025), with three patients who underwent emergency surgery dying within 30 days due to LCOS, hemorrhage event, and refractory heart failure, respectively. We finally collected surgical data from 48 patients because two patients were operated at outside hospitals and one patient had failed surgical repair. All the 48 patients underwent repair surgery under the condition of cardiopulmonary bypass support (CPB) and moderate hypothermia myocardial protection. After general anesthesia, the operation was performed through a median sternal incision. Among the 48 patients, 32 patients (66.7%) had the infarct area excised or excluded by the Daggett or Daivd conventional surgical approach. The remaining 16 (33.3%) patients received a modified surgery method named SurCOP with a 100% success rate. This new technology was first carried out by our hospital, which improved the traditional method of using bovine pericardial patch alone, combined a T-shaped PDA occluder and a slightly larger patch in the VSR site. SurCOP precisely released the sealing material, remain stable under persistent exposure to high left-to-right pressure gradient, prevent the remaining shunt after surgery (32). There were no significant differences in operation consuming time, surgical incision selection, surgical repair method, whether CABG performed at the same time etc. between the survival and death groups.

**Table 4:** Preoperative and intraoperative features of VSR patients in surgical repair group (*n* = 51).

**Preoperative and intraoperative characteristics**	**Grouping**	**Total number of cases** **(*n* = 51)**	**Survival group** **(*n* = 33)**	**Death group** **(*n* = 18)**	**χ^2^/t value**	***P* value**
Cardiogenic shock, [n (%)]		5 (9.8%)	0 (0.0%)	5 (27.8%)	7.264	**0.007**
NT-pro BNP, (pg/ml, $\bar{\chi }$ ± s)		9,391.7 ± 8,818.5	6,806.6 ± 6,824.9	13,843.8 ± 10,208.0	2.606	**0.015**
EuroSCORE II, ($\bar{\chi }$ ± s)		13.5 ± 2.7	12.1 ± 1.9	16.1 ± 1.9	7.036	**< 0.001**
Surgical status, [n (%)]	Emergency operation	18 (35.3%)	8 (24.2%)	10 (55.6%)	5.001	**0.025**
	Elective operation	33 (64.7%)	25 (75.8%)	8 (44.4%)		
Operation consuming time, (h, $\bar{\chi }$ ± s)		4.4 ± 1.2	4.2 ± 1.2	4.8 ± 1.3	1.475	0.147
Surgical incision, [n (%)]	Ventricular aneurysm	43 (89.6%)	28 (90.3%)	15 (88.2%)	2.391	0.813
	Left ventricular	1 (2.1%)	1 (3.2%)	0 (0.0%)		
	Right atrium	3 (6.3%)	2 (6.5%)	1 (5.9%)		
	Aortic root	1 (2.1%)	0 (0.0%)	1 (5.9%)		
Surgical repair method, [n (%)]	Traditional surgical approach	32 (66.7%)	21 (67.7%)	11 (64.7%)	0.046	0.831
	SurCOP	16 (33.3%)	10 (32.3%)	6 (35.3%)		
Concomitant CABG, [n (%)]		28 (58.3%)	20 (64.5%)	8 (47.1%)	1.377	0.241
Number of graft vessels, [n (%)]	1	18 (64.3%)	13 (61.9%)	5 (71.4%)	0.207	0.649
	2	10 (35.7%)	8 (38.1%)	2 (28.6%)		
Type of graft vessels, [n (%)]	SVG	19 (67.9%)	13 (61.9%)	6 (85.7%)	1.081	0.794
	LIMA	2 (7.1%)	2 (9.5%)	0 (0.0%)		
	SVG+LIMA	7 (25.0%)	6 (28.6%)	1 (14.3%)		
Concomitant ventricular aneurysm resection, [n (%)]		33 (68.8%)	22 (71.0%)	11 (64.7%)	0.200	0.654
Concomitant bicuspid annuloplasty, [n (%)]		3 (6.3%)	2 (6.5%)	1 (5.9%)	0.000	1.000
Concomitant tricuspid annuloplasty, [n (%)]		7 (14.6%)	6 (19.4%)	1 (5.9%)	0.701	0.402

^*^VSR, ventricular septal rupture; NT-pro BNP, N-terminal pro b-type natriuretic peptide; EuroSCORE II, European heart surgery risk assessment system II; SurCOP, surgical repair combining an occluder and a patch; CABG, coronary artery bypass grafting; SVG, saphenous vein graft; LIMA, left internal mammary artery. P values in bold meant significantly different (P < 0.05).

### Postoperative characteristics

Comparison of postoperative characteristics of VSR patients between surgical repair group and percutaneous TCC group are shown in Table 5. A total of four (11.8%) patients in the percutaneous TCC group failed in sealing, two because the guide wire could not pass through the ventricular septal defect to the right ventricle and pulmonary artery, one because the umbrella fixation failed after sealing, and one because the sealing device slipped into the right ventricle after repeated attempts. In the surgical repair group, one (2.0%) patient failed in sealing because of serious infarction and the risk of heart rupture. After analysis and comparison, we determined that the duration of ventilator use was longer in the surgical repair group [percutaneous TCC group 0.0 (0.0, 16.8) vs. surgical repair group 37.9 (20.1, 62.0), *P* < 0.001]. In addition, patients in the surgical repair group also had longer hospital stays [percutaneous TCC group (22.0±9.8) vs. surgical repair group (31.1 ± 17.0), *P* = 0.006]. The postoperative complications were: renal failure requiring CRRT (15,18.5%), postoperative blood transfusion (38, 46.9%) and LCOS (29, 35.8%). Compared with the percutaneous TCC group, the surgical repair group had a lower incidence of postoperative residual shunt [percutaneous TCC group (19, 57.6%) vs. surgical repair group (12, 24.5%), *P* = 0.002] but a higher incidence of postoperative blood transfusion [percutaneous TCC group (8, 24.2%) vs. surgical repair group (30, 62.5%), *P* = 0.001]. There was no significant difference in long-term mortality between the percutaneous TCC group and the surgical repair group (*P* = 0.779), but the percutaneous TCC group had a higher risk of readmission [percutaneous TCC group (15, 44.1%) vs. surgical repair group (8, 15.7%), *P* = 0.004] and higher odds of developing MACEs [percutaneous TCC group (26, 76.5%) vs. surgical repair group (25, 49.0%), *P* = 0.011]. The patients in this study were readmitted mainly due to angina pectoris, MI, or heart failure.

**Table 5:** Comparison of preoperative and postoperative characteristics of VSR patients between surgical repair group and percutaneous TCC group (*n* = 85).

**Preoperative and postoperative characteristics**	**Grouping**	**Total number of cases** **(*n* = 85)**	**Percutaneous TCC group** **(*n* = 34)**	**Surgical repair group** **(*n* = 51)**	**χ^2^/t value/Z value**	***P* value**
Cardiogenic shock, [n (%)]		11 (12.9%)	6 (17.6%)	5 (9.8%)	0.526	0.468
NT-pro BNP, (pg/ml, $\bar{\chi }$ ± s)		9,971.2 ± 11,335.7	10,806.3 ± 14,316.7	9,391.7 ± 8,818.5	0.557	0.579
Surgical status, [n (%)]	Emergency operation	27 (31.8%)	9 (26.5%)	18 (35.3%)	0.733	0.392
	Elective operation	58 (68.2%)	25 (73.5%)	33 (64.7%)		
Repair failure, [n (%)]		5 (5.9%)	4 (11.8%)	1 (2.0%)	1.992	0.158
Ventilator usage time, [h, M (P25, P75)]		22.7 (0.0, 52.9)	0.0 (0.0, 16.8)	37.9 (20.1, 62.0)	−5.058	**< 0.001**
Hospital stay, (d, $\bar{\chi }$ ± s)		27.5 ± 15.2	22.0 ± 9.8	31.1 ± 17.0	−2.804	**0.006**
ICU stay, (d, $\bar{\chi }$ ± s)		8.6 ± 10.3	10.6 ± 11.1	7.3 ± 9.6	1.465	0.147
Post-operative residual shunt, [n (%)]		31 (37.8%)	19 (57.6%)	12 (24.5%)	9.181	**0.002**
Postoperative CRRT, [n (%)]		15 (18.5%)	4 (12.1%)	11 (22.9%)	1.510	0.219
Postoperative blood transfusion, [n (%)]		38 (46.9%)	8 (24.2%)	30 (62.5%)	11.493	**0.001**
LCOS, [n (%)]		29 (35.8%)	10 (30.3%)	19 (39.6%)	0.733	0.392
EuroSCORE II, ($\bar{\chi }$ ± s)		13.7 ± 2.6	13.9 ± 2.4	13.5 ± 2.7	0.762	0.448
Readmission, [n (%)]		16 (18.8%)	11 (32.4%)	5 (9.8%)	6.788	**0.009**
30-day mortality, [n (%)]		24 (28.2%)	10 (29.4%)	14 (27.5%)	0.039	0.844
MACEs, [n (%)]		40 (47.1%)	21 (61.8%)	19 (37.3%)	4.919	**0.027**
Overall mortality, [n (%)]		29 (34.1%)	11 (32.4%)	18 (35.3%)	0.079	0.779

^*^VSR, ventricular septal rupture; TCC, transcatheter closure; NT-pro BNP, N-terminal pro b-type natriuretic peptide; ICU, intensive care unit; CRRT, continuous renal replacement therapy; LCOS, low cardiac output syndrome; EuroSCORE II, European heart surgery risk assessment system II; MACE, major adverse cardiac events. P values in bold meant significantly different (P < 0.05).

### The long-term mortality

**Figure 2:**
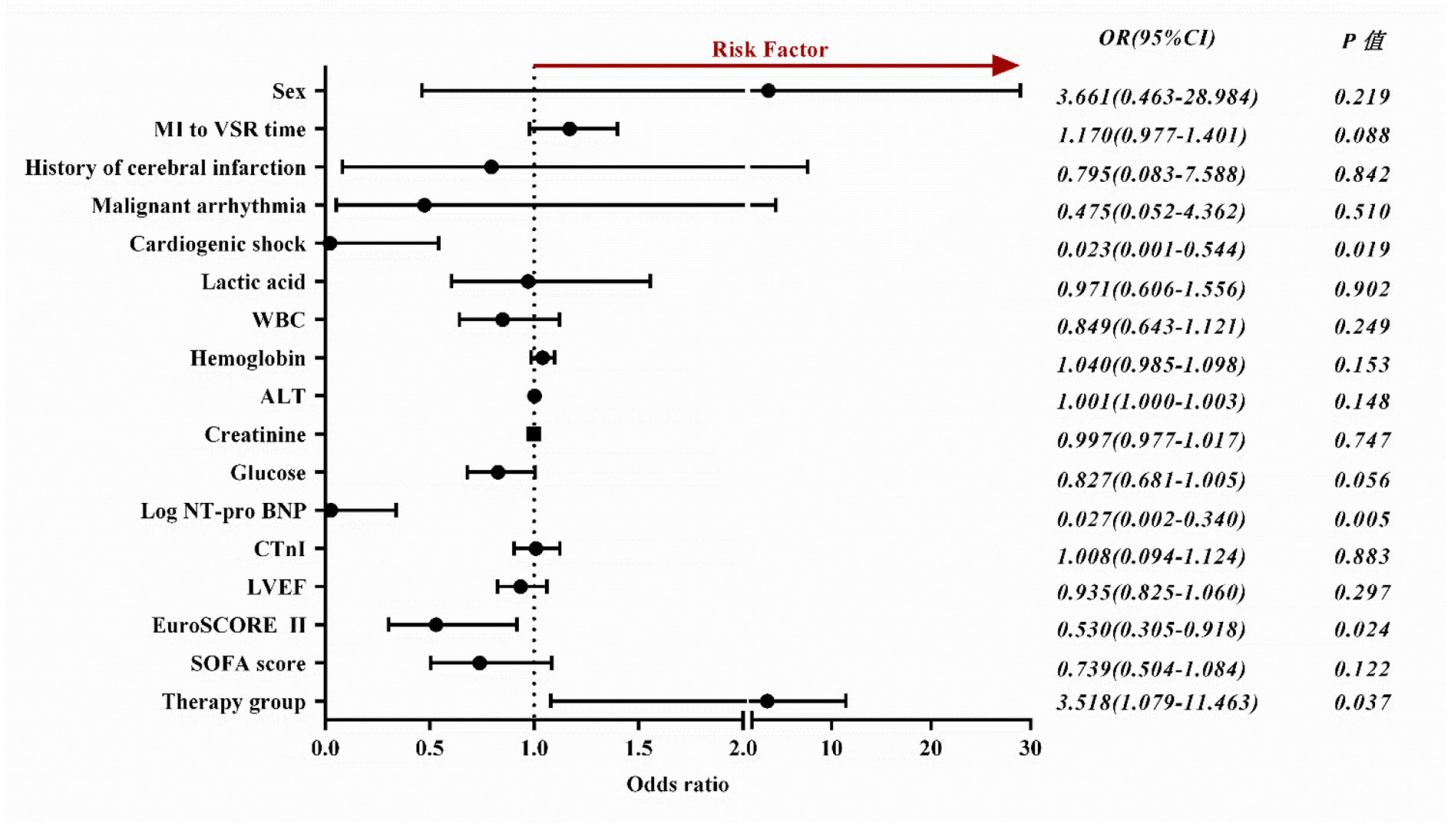
Forest plot depicts factors influencing the long-term prognosis of patients with VSR. OR, odds ratio; CI, confidence interval; MI, myocardial infarction; VSR, ventricular septal rupture; WBC, white blood cells; ALT, alanine transaminase; NT-pro BNP, n-terminal pro b-type natriuretic peptide; CTnI, cardiac troponin I; LVEF, left ventricle ejection fraction; SOFA, sequential organ failure assessment.

Binary Logistic regression showed that cardiogenic shock (odds ratio [OR], 0.023; 95% CI, 0.001–0.544; *P* = 0.019), high levels of NT-pro BNP (OR, 0.027; 95% CI, 0.002–0.340; *P* = 0.005), high EuroSCORE II score (OR, 0.530; 95% CI, 0.305–0.918; *P* = 0.024) and conservative medical treatment (OR,3.518; 95% CI, 1.079–11.463; *P* = 0.037) were independently associated with long-term mortality. In order to exclude the bias caused by the severity of the disease in the medication group, we analyzed the patients in the non-medication group separately and found that the above indicators were still significant (Supplementary Table 1). ORs with 95% CIs are presented in Figure 2.

**Figure 3:**
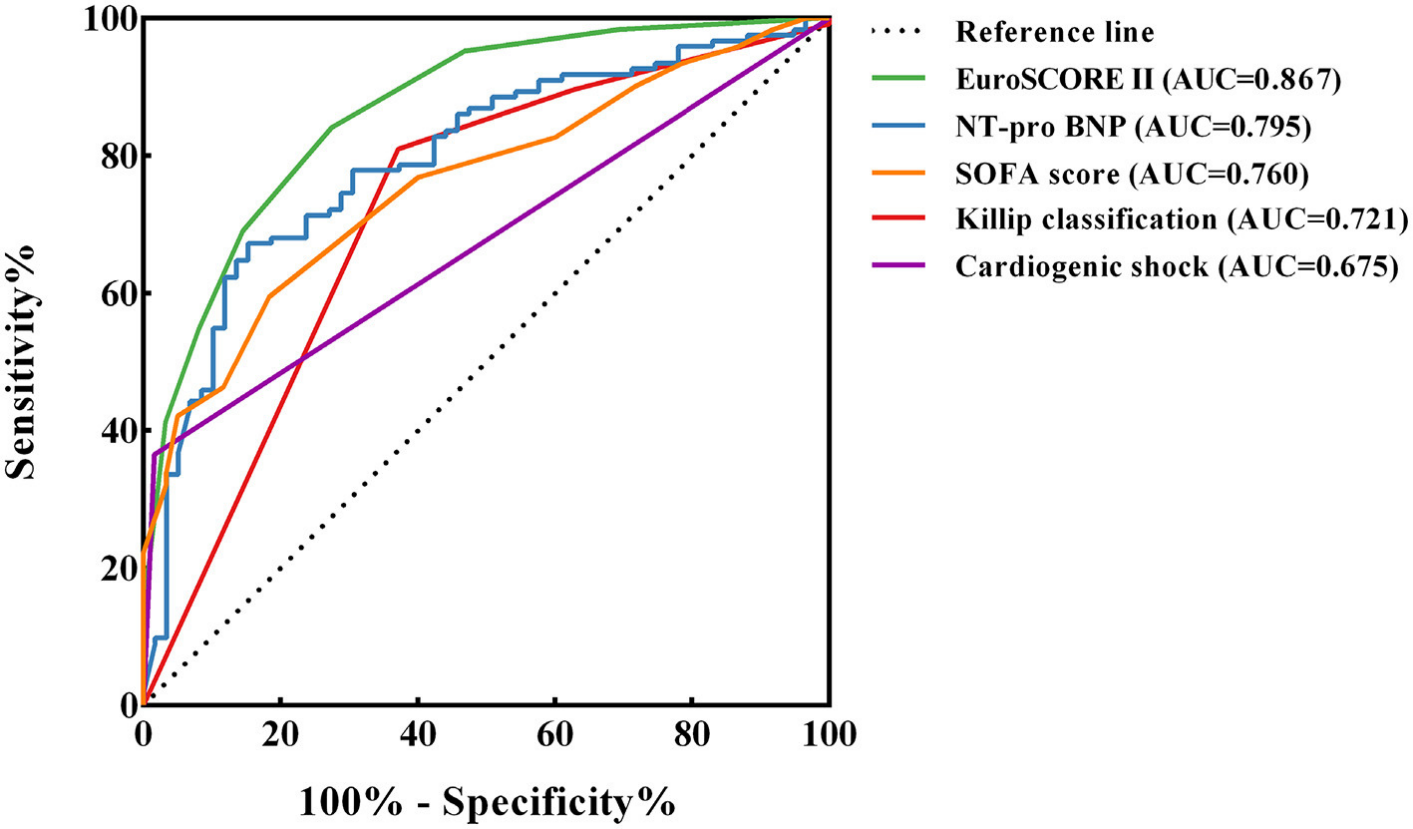
ROC curves of EuroSCORE II, NT-pro BNP, SOFA score, Killip classification and Cardiogenic shock. ROC, receiver operating characteristic; AUC, area under curve ROC; CI, confidence interval; NT-pro BNP, n-terminal pro b-type natriuretic peptide; SOFA, sequential organ failure assessment. The following are the details of the five ROC curves: EuroSCORE II: Cut-off value = 14; AUC = 0.867 (95%CI: 0.813–0.921, *P* < 0.001); Sensitivity: 84.1%; Specificity: 72.6% NT-pro BNP: Cut-off value = 8,091; AUC = 0.795 (95%CI: 0.726–0.863, *P* < 0.001); Sensitivity: 67.2%; Specificity: 84.7% SOFA score: Cut-off value = 13; AUC = 0.760 (95%CI: 0.690–0.829, *P* < 0.001); Sensitivity: 59.5%; Specificity: 81.7% Killip classification: AUC = 0.721 (95%CI: 0.639–0.802, *P* < 0.001); Sensitivity: 81.0%; Specificity: 62.9% Cardiogenic shock: AUC = 0.675 (95%CI: 0.599–0.750, *P* < 0.001); Sensitivity: 36.5%; Specificity: 98.4%.

**Figure 4:**
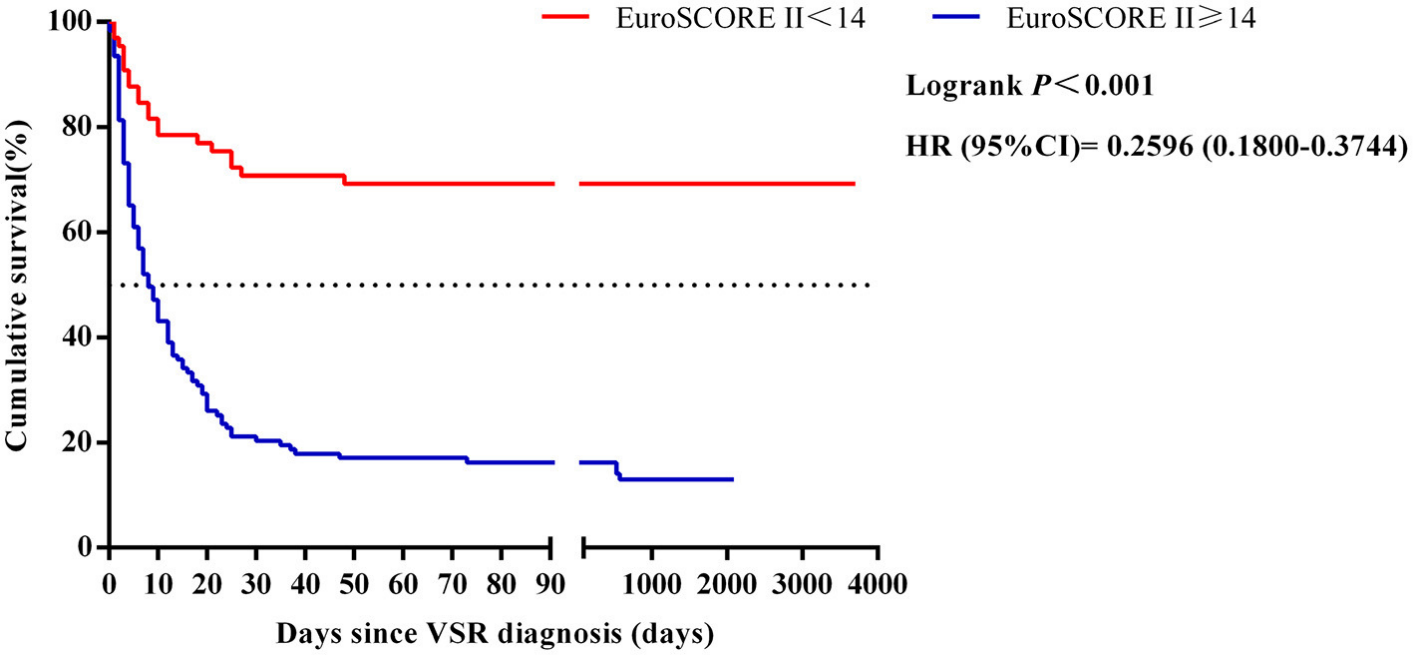
Relationships between the EuroSCORE II scores and 10-year mortality in patients with VSR. HR, hazard ratio; CI, confidence interval; VSR, ventricular septal rupture.

The ROC curves for EuroSCORE II, NT-pro BNP, SOFA score, Killip classification and cardiogenic shock were plotted separately to evaluate their predictive power for long-term mortality, yielding an AUC of 0.867, 0.795, 0.760, 0.721 and 0.675, respectively. The specific results are shown in Figure 3 and the legend. The results indicated that EuroSCORE II predicted long-term mortality with high accuracy. The cut-off point was determined to be 14 by calculating the maximum of the Youden's index. According to the cut-off point, the patients were divided into EuroSCORE II < 14 group and EuroSCORE II ≥ 14 group. The median survival time estimates for EuroSCORE II < 14 group and EuroSCORE II ≥ 14 group were 681.3 days and 8.0 days, respectively. The survival curves of were plotted using the Kaplan-Meier method and Log-rank test showed in Figure 4 and there were statistically significant differences between EuroSCORE II < 14 group and EuroSCORE II ≥ 14 group (HR = 0.2596, 95%CI: 0.1800–0.3744, Logrank *P* < 0.001).

Supplementary Figure 1 reflects the relationship between timing of surgery and mortality in the surgical repair group (*P* = 0.058).

## Discussion

In this single center retrospective study of 188 patients, we explored the factors influencing the long-term prognosis of patients with VSR from several aspects and evaluated the performance of scoring systems such as EuroSCORE II. Overall, long-term mortality was higher in women, patients with previous myocardial infarction, malignant arrhythmias, cardiogenic shock, cardiac arrest, renal failure, and MODS (*P* < 0.05). In addition, these patients had a shorter MI to VSR time and a higher Killip classification than the survival group (*P* < 0.05). From the laboratory data, there were also significant differences in lactic acid, white blood cell, NT-pro BNP and other indicators between the death group and the survival group (*P* < 0.05). According to the results of TTE, the LVEF in the death group was lower (*P* < 0.05), and the perforation location was also related to the mortality. Compared with the apical and anterior VSR, the mortality of patients with posterior VSR was lower. In terms of MI territory, the prognosis of extensive anterior MI was worse, although there was no significant difference in this study (*P* = 0.093), an anterior MI is more prone to VSR, and tend to produce simple apical defects. In contrast, inferior or lateral MIs are more likely to result in extensive and irregular basal defects (5, 33, 34). Furthermore, the timing of surgical repair also affects the long-term survival of VSR patients. In this study, the survival rate of patients who underwent repair after 2 weeks of septal perforation was greatly improved, including those who used a mechanical circulatory support to extend the timing of surgery beyond 2 weeks (*P* < 0.05). Our study showed that women, worse Killip classification and cardiogenic shock were risk factors for long-term mortality in VSR patients, which is consistent with many existing studies (3, 35, 36). However, due to differences in the study cohort, age did not have a significant effect on survival in patients with VSR in this study.

We also compared the effects of medical treatment, percutaneous TCC and surgical repair on the long-term prognosis of patients. The results showed that the mortality of patients in the medical conservative treatment group was much higher than that in the other two groups. However, there is also a survival bias caused by conservative treatment due to unstable hemodynamics in some patients. The prognosis of these patients with early hemodynamic instability is often worse (15). At present, there is no uniform standard for the optimal period of surgical repair. The 2013 AHA guidelines for STEMI recommended emergency surgical repair for all patients with VSR, even in hemodynamically stable patients (11). However, due to an inadequate transportation system in middle-income economies like China, a large proportion of VSR patients cannot receive emergency surgery in a timely manner and therefore, unplanned delayed surgery is common in China (3).

Ronco D et al. found that delayed surgery appeared to be associated with better survival, while emergent surgery was associated with a higher mortality (37). According to the Society of Thoracic Surgeons (STS) database, the mortality rate for patients who underwent surgery within 7 days of septal perforation was 54.1%, while the mortality rate for patients who underwent surgery after 7 days was 18.4% (38). Therefore, theoretically the best surgical timing is 2 weeks after septal perforation and in a hemodynamically stable state. Unrestricted delayed surgery is not advocated because of the possibility of enlarging interventricular communication, which could increase the shunt fraction and worsen right ventricular overload. There are usually only small single-center studies in the literature, but few large international multicenter studies, thus limiting the evidence in this area (16, 37). Malik J et al. concluded that one way to improve survival outcomes in patients with VSR is to use mechanical circulatory support to assist in delaying surgery (39).

IABP, implantable turbine-pump and percutaneous cardiopulmonary bypass support are different cardiac assist devices commonly used today to stabilize patients with AMI (40). Indeed, the characteristics of maintaining preoperative patient stability and protecting the early perioperative course have led to the increasing acceptance of MCS in the treatment of patients with VSR by a growing number of physicians (16, 41, 42). In this study, the proportion of using IABP, CRRT and CVC in the death group is higher than that in the survival group, and most of the death group patients who rely on IABP support are difficult to go offline, which also shows that unlimited delayed surgery may cause unnecessary patients' pain and costs, especially for those relaying on mechanical assist. Therefore, in hemodynamically unstable VSR patients, the best timing of delayed surgery is that patients receive surgery intervention as soon as they reach healed phase (43).

Additional operations for surgical repair of VSR including CABG operation in 28 patients, ventricular aneurysm resection in 33 patients, bicuspid annuloplasty in 3 patients and tricuspid annuloplasty in seven patients, which showed no difference between the death group and the survival group. There is no clear conclusion about the pros and cons of these additional procedures. In the case of CABG, Abu-Omar et al. reported that CABG did not improve long-term prognosis, but rather prolonged the procedure and increased the risk of surgery for patients (44). However, some studies have also suggested that CABG be performed in conjunction with perforation repair (45). Studies have shown that the advantages of left ventricular aneurysm resection in reducing ventricular volume, restoring ventricular shape, and reducing the arrhythmic risk related to necrotic scar, have been well addressed in the past decades, but it is also possible that the patient cohort considered in the paper is somehow different from the patients generally presented and managed in other studies (38, 46, 47).

Percutaneous TCC has have become increasingly sophisticated and can be used as an alternative to surgical closure of VSR for subacute and chronic VSR (48). When applied in patients with advanced VSR is usually associated with a high mortality rate (2).

In this study, the postoperative characteristics of VSR patients in the surgical repair group and the percutaneous TCC group were compared from the aspects of hospital stay, post-operative residual shunt and so on. The ventilator usage time, hospital stay, and the probability of postoperative hemolysis in the surgical repair group were higher than those in the percutaneous TCC group, and the latter had a higher probability of post-operative residual shunt and readmission than the surgical repair group. The rate of MACEs was higher in the percutaneous TCC group. Currently, the comparison between surgical repair and percutaneous TCC for the treatment of VSR is limited, and more large-sample clinical studies are needed to further compare the advantages and disadvantages of the two methods.

EuroSCORE was originally a quantitative risk assessment system used to predict in-hospital mortality after cardiovascular surgery (49). With the increase in relevant studies, its latest version, EuroSCORE II, is widely used for preoperative risk assessment in cardiac surgery and is part of routine care in many cardiac centers (50–52). In this study, three commonly used scoring systems, Gensini score, SOFA score and EuroSCORE II, were selected to analyze and compare their ability to predict the long-term prognosis of VSR patients. The results show that the prediction accuracy of EuroSCORE II for long-term mortality is much higher than the other two scores. This shows that EuroSCORE II can be used not only for preoperative risk assessment of cardiac surgery, but also as one of the risk assessment factors for the prognosis of VSR patients. In this study, combined cardiogenic shock and higher NT-pro BNP levels were strongly associated with high mortality in VSR patient and were not affected by treatment grouping. If these two items are added to the original EuroSCORE II score, the predictive accuracy may be further improved, which also needs to be confirmed by a large number of related studies.

This study explored factors affecting the long-term prognosis of patients with VSR and evaluated multiple scoring systems, such as EuroSCORE II, in a comprehensive manner. This study has its limitations. First, because surgery is usually performed in patients with relatively stable VSR, it is inevitable that these patients may have a better prognosis than patients with early hemodynamic instability. Second, this study is a single-center, small-sample study, and further validation by a multicenter, large-sample prospective randomized controlled trial is needed to allow further generalization of the findings.

## Conclusion

In this study, the mortality of patients in the medication group was much higher than that in the surgical repair group and percutaneous TCC group. Moreover, we found that whether combined with cardiogenic shock, NT-pro BNP level, EuroSCORE II score and treatment mode were independently associated with long-term mortality in patients with VSR. From the perspective of postoperative characteristics, surgical repair and percutaneous TCC have their own advantages and disadvantages, which still need to be further explored by multi center and large sample research. EuroSCORE II score has a better predictive ability for long-term mortality in VSR patients, it can be extended by adding combined cardiogenic shock and NT-pro BNP levels to the original score. Two weeks after ventricular septal perforation is an appropriate time for surgical occlusion, which can better improve the prognosis and reduce mortality in patients with VSR.

## Data availability statement

The original contributions presented in the study are included in the article/Supplementary material, further inquiries can be directed to the corresponding author.

## Ethics statement

The studies involving human participants were reviewed and approved by Ethics Committee of Zhengzhou University. The patients/participants provided their written informed consent to participate in this study. Written informed consent was obtained from the individual(s) for the publication of any potentially identifiable images or data included in this article.

## Author contributions

M-XD, S-LL, and Z-ZY: software, formal analysis, data curation, and writing—original draft. XZ, J-ZT, and B-YL: conceptualization, methodology, and writing—review and editing. X-GM, D-PD, Y-YL, and YD: validation and formal analysis. SP, L-PB, and Q-YZ: investigation. Z-AR, Y-HZ, and G-RM: supervision. X-YZ: term, resources, project administration, and funding acquisition. All authors contributed to the article and approved the submitted version.

## Funding

This study was partly supported by Joint Project of Medical Science and Technology of Henan (LHGJ20190095) awarded to XZ and Program for Science and Technology Development of Henan Province (212102310210) awarded to X-YZ and XZ puts forward ideas, designs experiment and modifies paper. X-YZ supervises and guides the team, and decides to submit the article for publication.

## Conflict of interest

The authors declare that the research was conducted in the absence of any commercial or financial relationships that could be construed as a potential conflict of interest.

## Publisher's note

All claims expressed in this article are solely those of the authors and do not necessarily represent those of their affiliated organizations, or those of the publisher, the editors and the reviewers. Any product that may be evaluated in this article, or claim that may be made by its manufacturer, is not guaranteed or endorsed by the publisher.
