# Supplementary material for: Analysis of influencing factors for prognosis of patients with ventricular septal perforation: A single-center retrospective study
Source: Front Cardiovasc Med. 2022 Nov 3;9:995275. doi: 10.3389/fcvm.2022.995275 (PMC9668866; doi:10.3389/fcvm.2022.995275)
Supplement: Supplementary file 1 [file Data_Sheet_1.pdf]

Supplementary table 1. Further analysis of factors influencing the survival rate of patients in the non-pharmacological treatment group. (n=85)

Clinical data	Grouping	Total number of cases (n=85)	Survival group (n=56)	Death group (n=29)	χ^2/t value/Z value	*P* value
Sex, [n (%)]	Male	53 (62.4%)	38 (67.9%)	15 (51.7%)	2.118	0.146
	Female	32 (37.6%)	18 (32.1%)	14 (48.3%)		
MI to VSR time, [d, M (P25, P75)]		5.0 (2.0, 10.0)	5.5 (1.3, 10.0)	4.5 (3.0, 7.0)	-1.101	0.271
History of cerebral infarction, [n (%)]		10 (11.8%)	5 (8.9%)	5 (17.2%)	0.597	0.440
Malignant arrhythmia, [n (%)]		16 (18.8%)	5 (8.9%)	11 (37.9%)	10.517	**0.001**
Cardiogenic shock, [n (%)]		5 (9.8%)	0 (0.0%)	5 (27.8%)	7.264	**0.007**
Lactic acid, [mmol/L, M (P25, P75)]		1.7 (1.1, 2.6)	1.5 (0.9, 2.1)	2.0 (1.5, 4.8)	-2.585	**0.010**
WBC, [*10^9/L, M (P25, P75)]		9.3 (6.9, 13.2)	8.7 (6.6, 12.1)	11.4 (9.1, 15.2)	-3.692	**<0.001**
Hemoglobin, (g/L, χ ±s)		127.1±18.4	128.5±17.9	124.4±19.3	-0.958	0.341
ALT, [U/L, M (P25, P75)]		35.5 (20.3, 159.5)	32.5 (16.3, 71.3)	159.0 (42.0, 701.0)	-3.778	**<0.001**
Creatinine, [μmol/L, M (P25, P75)]		90.6 (69.6, 129.0)	86.5 (68.0, 103.6)	127.5 (87.8, 213.3)	-3.430	**0.001**
Glucose, [mmol/L, M (P25, P75)]		6.8 (5.2, 9.2)	6.4 (5.1, 8.2)	8.0 (6.1, 11.8)	-1.501	0.133

NT-pro BNP, [pg/ml, M (P25, P75)]	5554.3 (3230.0, 14422.0)	4543.5 (2235.1, 7250.7)	15000.0 (6105.5, 24578.8)	-4.661	**<0.001**
CTnI, [mmol/L, M (P25, P75)]	0.6 (0.1, 2.0)	0.3 (0.0, 2.6)	0.9 (0.6, 3.0)	-2.431	**0.015**
LVEF, (%, $\bar{\chi}$±s)	51.1±10.2	51.1±10.0	51.3±10.6	0.095	0.924
EuroSCORE II, ($\bar{\chi}$ ±s)	13.7±2.6	12.5±1.9	15.9±2.1	7.553	**<0.001**
SOFA score, ($\bar{\chi}$ ±s)	11.6±4.4	10.0±3.1	14.5±5.0	4.393	**<0.001**

*****MI, myocardial infarction; VSR, ventricular septal rupture; WBC, white blood cells; ALT, alanine transaminase; NT-pro BNP, N-terminal pro b-type natriuretic peptide; CTnI, cardiac troponin I; LVEF, left ventricle ejection fraction; EuroSCORE II, European heart surgery risk assessment system II; SOFA, sequential organ failure assessment. *P* values in bold meant significantly different ($P<0.05$).

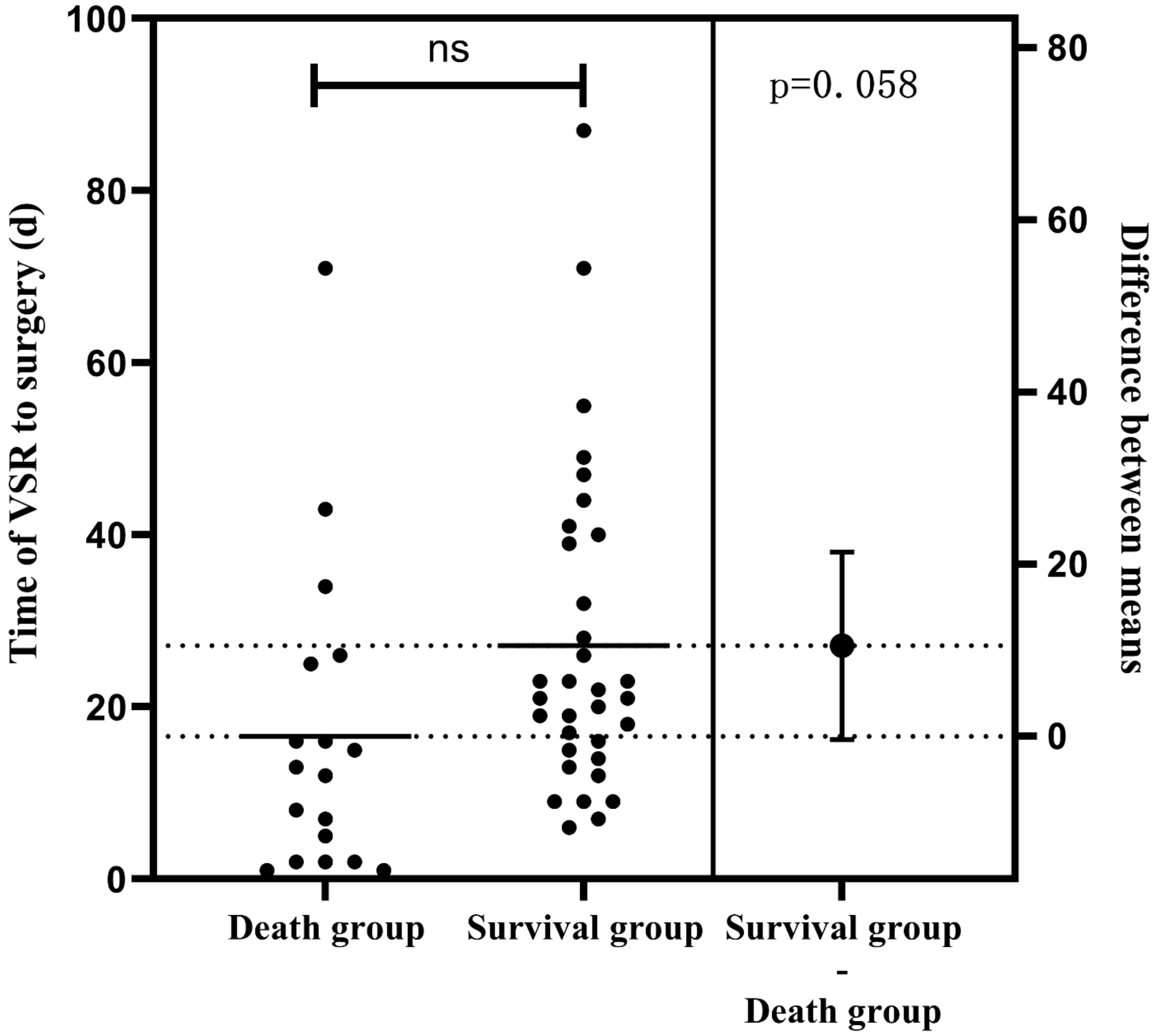

Supplementary Figure 1. Estimation chart of the relationship between surgery timing and survival rate in surgical repair group.

ns: no significance
